# Mitochondrial DNA Variability within *Uromastyx ornata philbyi* (Agamidae: Squamata) from Southwestern Saudi Arabia

**DOI:** 10.1155/2012/851379

**Published:** 2012-01-19

**Authors:** Sayed A. M. Amer, Mohamed M. Ahmed, Thomas M. Wilms, Mohammed Shobrak, Yoshinori Kumazawa

**Affiliations:** ^1^Department of Biotechnology, Faculty of Science, Taif University, Taif, P.O. Box 888, Saudi Arabia; ^2^Department of Zoology, Faculty of Science, Cairo University, Giza, Egypt; ^3^Department of Biochemistry, Faculty of Veterinary Medicine, Minufiya University, Sadat City, Egypt; ^4^Zoologischer Garten Frankfurt, Bernhard-Grzimek-Allee 1, 60316 Frankfurt am Main, Germany; ^5^Department of Biology, Faculty of Science, Taif University, Taif, P.O. Box 888, Saudi Arabia; ^6^Graduate School of Natural Sciences, Nagoya City University, Aichi 467-8501, Japan

## Abstract

Approximately 2.4 kbp of mitochondrial DNA was sequenced from 9 individuals of *Uromastyx ornata philbyi* originating from Taif, Namas, Al-Baha, and Jazan in southwestern Saudi Arabia. The sequenced regions cover eight tRNA genes (tRNA^Gln^, tRNA^Ile^, tRNA^Met^, tRNA^Trp^, tRNA^Ala^, tRNA^Asn^, tRNA^Cys^, and tRNA^Tyr^) and two protein-coding genes (NADH dehydrogenase subunit 2 and cytochrome *b*). *U. ornata philbyi* had an insertion of 170 bp length between tRNA^Gln^ and tRNA^Ile^ genes. The first 128 bp of this insertion was similar to the one identified earlier in *U. ornata ornata* and can be folded into a stem-and-loop structure, which was less stable in *U. ornata philbyi* than in *U. ornata ornata*, or the second tRNA^Gln^ gene. The next 42 bp of the insertion was unique in *U. ornata philbyi* and additionally retained a stable stem-and-loop structure. Most base substitutions found in the sequenced genes were synonymous transitions rather than transversions. Tree analyses supported the sister group relationship between the two *U. ornata* subspecies and divided *U. ornata philbyi* into two groups: Taif+Namas group in the east of Sarawat and Al-Baha+Jazan group in the west of Sarawat. These molecular data are in agreement with current classification of *U. ornata*.

## 1. Introduction

Two subspecies of *Uromastyx ornata* have been recognized: *Uromastyx ornata ornata *(Heyden 1827) and *Uromastyx ornata philbyi *Parker, 1938 [1]. The distribution of *U. ornata* is confined to the eastern vicinity of the Red Sea. The range includes the Sinai Peninsula in the north and extends to the south east to Aqaba and southward through western Saudi Arabia to the northern border of Yemen. The presumed distribution of *Uromastyx ornata philbyi* extends along the mountainous region of the western Arabian Peninsula from Aqaba in the north down to Yemen. This area extends for about 1700 km through the arid and semiarid mountain range close to the coast of the Red Sea. The known geographical distribution includes the Median Mountains extending to the north, the Hijaz Mountains extending to the west of Makkah, and the Sarawat Mountains extending from west of Makkah down to the Yemen borders. *Uromastyx o. ornata *occurs on the Sinai Peninsula and northwestern Saudi Arabia as well as at the extreme southern tip of Palestine. 

 Wilms and Böhme [2] discussed the overlapping distribution of the two subspecies. The authors found that occurrence of both phenotypes broadly overlaps in the mountains of western Saudi Arabia, based on specimens of *U. o. ornata* from inside the range of *U. o. philbyi* at the vicinity of Jeddah. These localities are 800–1300 km further south from the type locality of *U. o. ornata*. According to Arnold [3], the northernmost locality of *U. o. philbyi *is not far from the type locality of *U. o. ornata*. In Jeddah and Jazan both phenotypes are obviously sympatric [2].

 Based on the specimens available, a final decision on the taxonomic status of *U. o. philbyi *is not yet possible. Wilms and Böhme [7] suggested three hypotheses to deal with this taxonomic problem. (1) Both taxa (*U. o. ornata *and *U. o. philbyi*) are part of an intraspecific cline: *U. o. philbyi *had to be synonymised with *U. o. ornata*. (2) There are no intermediate specimens in the region in question (no gene flow between the two sympatric phenotypes): *U. o. philbyi *had to be lifted to specific rank. (3) There is a secondary contact zone with gene flow between the two types: *U. o. philbyi *must be treated as a subspecies of *Uromastyx ornata*. The authors concluded that none of these hypotheses can be excluded with certainty and suggested that until more specimens and/or other data are available, *U. o. philbyi *had to be treated as a subspecies of *U. ornata*. The present study therefore aimed to highlight the molecular variability within *U. o. philbyi* and its relation to *U. o. ornata* to address its taxonomic status.

## 2. Material and Methods

**Figure 1:**
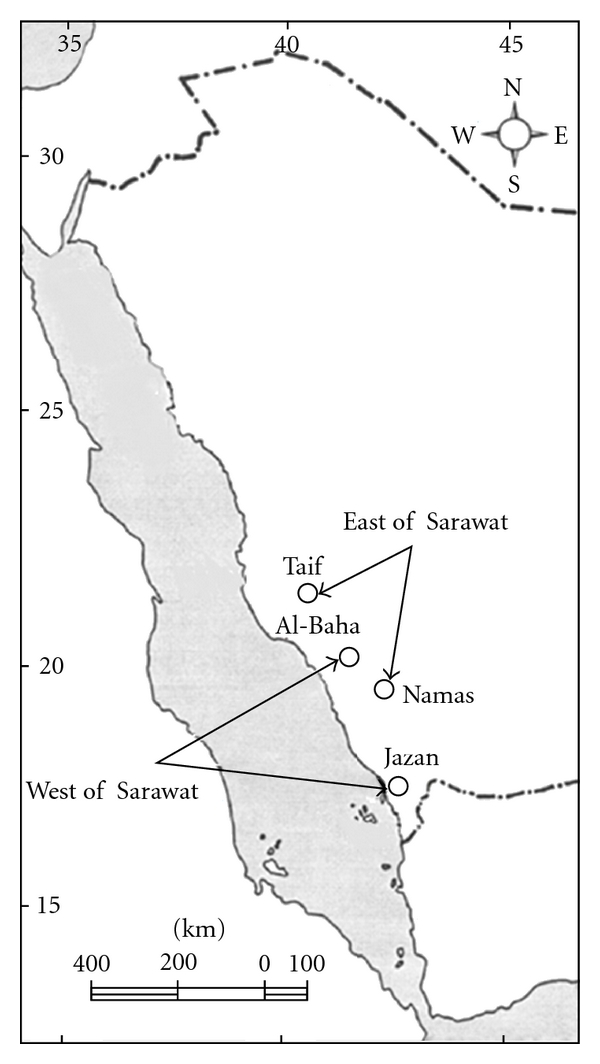
A map with southwestern Saudi Arabian localities (indicated as circles) from which samples of *U. o. philbyi* were collected.

Nine specimens of *U. o. philbyi *were collected from four localities on either side of the Sarawat Mountains in southwestern Saudi Arabia. These localities are Taif, Namas, Al-Baha, and Jazan (Figure 1). The collected animals were 3 specimens from Namas and 2 from each of the other localities. Individuals were taken to the lab, euthanized, and dissected. Samples of blood and muscle tissues were taken and immediately frozen at −80°C until use. 

DNA was extracted from 0.5 mL blood samples with EZ-10 Spin Column Genomic DNA MiniPreps Kit according to the manufacturer's instruction. Extracted DNA was spectrophotometrically quantified at 260/280 nm and was used for polymerase chain reaction (PCR).

Polymerase chain reaction was set up in a total volume of 10 *μ*L that contained 1.0 *μ*L of 10X Fast buffer, 0.8 *μ*L of 2.5 *μ*M dNTPs, 1.0 *μ*L each of 10 *μ*M primers, 0.05 *μ*L of SpeedStar HS (5 unit/uL) DNA polymerase (Takara Shuzo Co., Japan), and approximately 1 ng/mL of total genomic DNA. PCR was carried out with a Takara thermal cycler using the following cycling program: 32 cycles of denaturation (98°C, 5 s), annealing (55°C, 15 s), and extension (72°C, 20 s). After treatment with ExoSAP-It (Amersham, code US78200) at 37°C, the PCR products were sequenced on an automated DNA capillary sequencer (ABI 3500 Genetic Analyzer with 8 capillaries; Applied Biosystems) with amplification primers, using the BigDye Terminator v3.1 Cycle Sequencing Kit (Life Technologies). The sequencing reactions consisted of 25 cycles of 96°C for 10 s, 50°C for 5 s, and 60°C for 4 min. The positions and sequences of the primers that have been used in both amplifications and sequencing are listed in Table 1.

Nucleotide sequences of the mitochondrial ND2 region (the complete NADH dehydrogenase subunit 2 gene plus 8 flanking tRNA genes) were aligned with those obtained from the DDBJ database (3 *U. o. ornata*, 3 *U. ocellata,* and 1 *U. benti*). Two additional outgroup taxa (*U. a. aegyptia* and *U. a. microlepis*) were also included in the alignment. The alignment was carried out by using the DNASIS 3.5 (Hitachi) and MacClade 4.03 (Sinauer Associates, Inc.) with manual adjustments. All unalignable and gap-containing sites were excluded from analyses so that 1504 sites were left for constructing the relationship. We primarily conducted the tree analyses by maximum-parsimony (MP), neighbor-joining (NJ), and maximum-likelihood (ML) methods. These analyses were done in PAUP* 4.0b10 [8] by heuristic searches with the TBR branch swapping, 10 random taxon additions, and 1000 bootstrap replications for each. We also run Bayesian (MrBayes version 3.0) analysis [9] with conditions similar to those published by Amer and Kumazawa [10] and Kumazawa [11].

## 3. Results and Discussion

Approximately 1750 bp from ND2 and 8 tRNA genes and 700 bp from cytb gene were sequenced for *U. o. philbyi* in this study. The genes sequenced in this study were deposited in the GenBank database (accession numbers AB641364-AB641383). The 8 tRNA genes represent two clusters which flank the ND2 gene from either side. These clusters are QIM cluster (the first three genes) and WANCY cluster (the next 5 genes).

**Figure 2:**
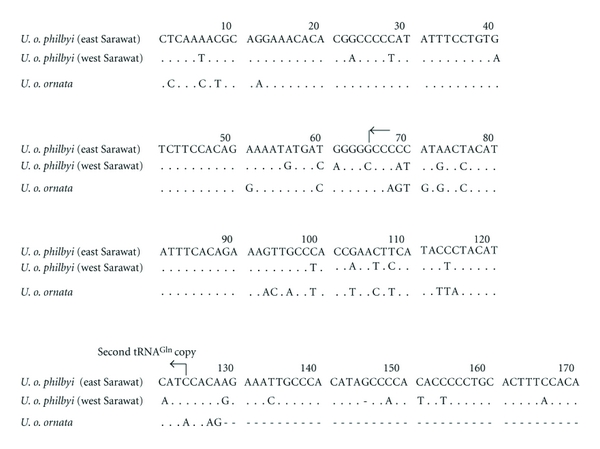
Aligned nucleotide sequences of the inserted region between the tRNA^Gln^ and tRNA^Ile^  genes in *U. o. philbyi* (east and west of Sarawat Mountains) and *U. o. ornata*. The extra bases (from 129 to 170) were found only in *U. o. philbyi*. Sequences are shown for the light strand. Dots indicate identity with the first sequence, and dashes denote a gap.

**Figure 3:**
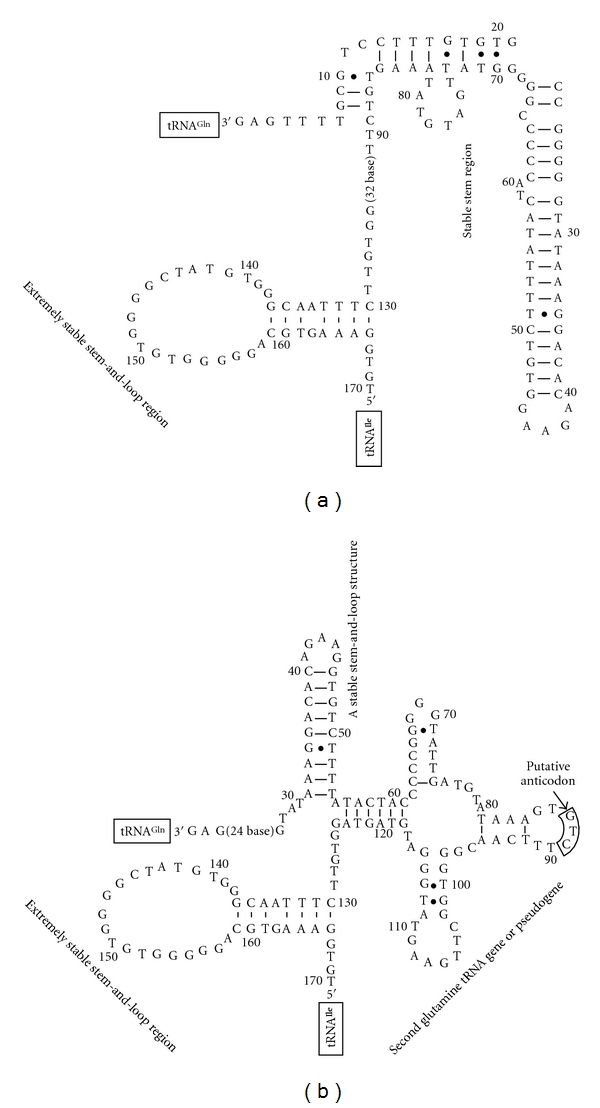
Secondary structures of the inserted sequences found between tRNA^Gln^ and tRNA^Ile^ genes in *U. o. philbyi *(represented by east Sarawat sequence). The first 128 bp insert can assume alternative secondary structures either with a moderately stable and long stem region (a) or with a clover-leaf structure for the second tRNA^Gln^ gene and a stable stem-and-loop structure (b). The next bolded 42 bp also assume a stable stem-and-loop structure. Heavy-strand sequences are shown, and numbers refer to the corresponding positions in their light-strand sequences of Figure 2. Bars in stems represent Watson-Crick base pairs, and dots stand for wobble G-U pairs for RNA.

 All available individuals from *U. o. philbyi* possessed an insertion of 170 bp between  tRNA^Gln^ and tRNA^Ile^ genes, and the first 128 bp of this insertion was similar to the one that was previously found for *U. o. ornata *[12] (Figure 2). This insertion assumed a less stable stem-and-loop secondary structure compared to that of *U. o. ornata* (Figure 3(a)). However, the remaining 42 bp of the insertion were only found in *U. o. philbyi* and exhibited a very strong stem-and-loop structure (Figures 3(a) and 3(b)). The 128 bp insertion showed 78% sequence identity between *U. o. ornata* and *U. o. philbyi* (base difference = 25 and 24 sites between *U. o. ornata* and both *U. o. philbyi*-East and *U. o. philbyi*-West, resp.) and 90% identity between the subpopulations of *U. o. philbyi *on either side of the Sarawat Mountains. The remaining part of the insertion (42 bp) showed 81% similarity between the haplogroups of east and west of the Sarawat Mountains. We searched for any similarity of the 42 bp in the vicinity of the sequenced genes and we did not find it. As revealed for *U. o. ornata*, the inserted sequence (from base 65 to base 124) could be folded into a clover-leaf structure to produce another copy of the tRNA^Gln^  gene or pseudogene (Figure 3(b)). This clover-leaf structure was more stable in *U. o. ornata* than in *U. o. philbyi* as the baseparing in the anticodon stem, D-stem and TΨC-stem was very weak in the latter by acquiring more wobble parings.

**Figure 4:**
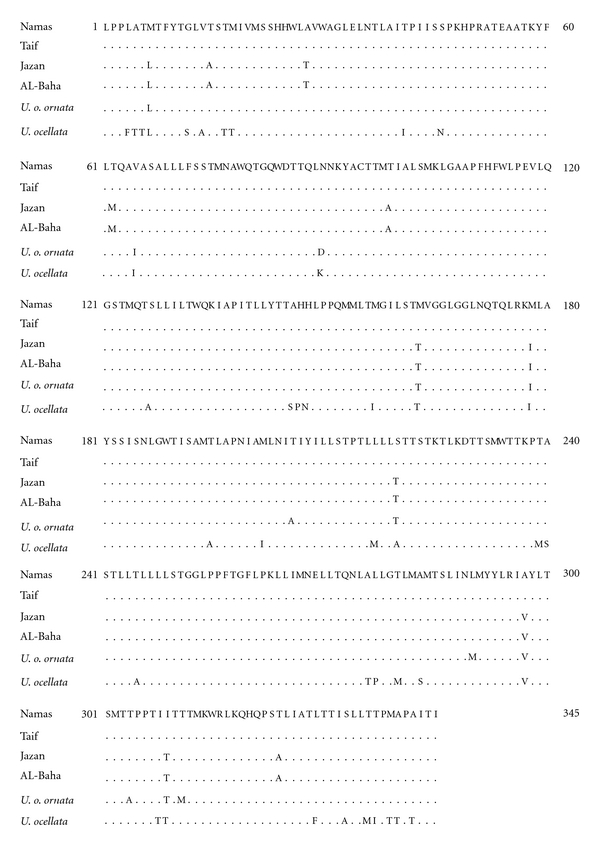
Aligned amino acid sequences of ND2 gene for *U. o. philbyi* from different localities. Complete amino acid sequence is given only to the ND2 of *U. o. philbyi* from Namas (east Sarawat), and dots for the other sequences denote the identity to this sequence. The sequence of *U. o. ornata* and *U. ocellata* are also included.

**Figure 5:**
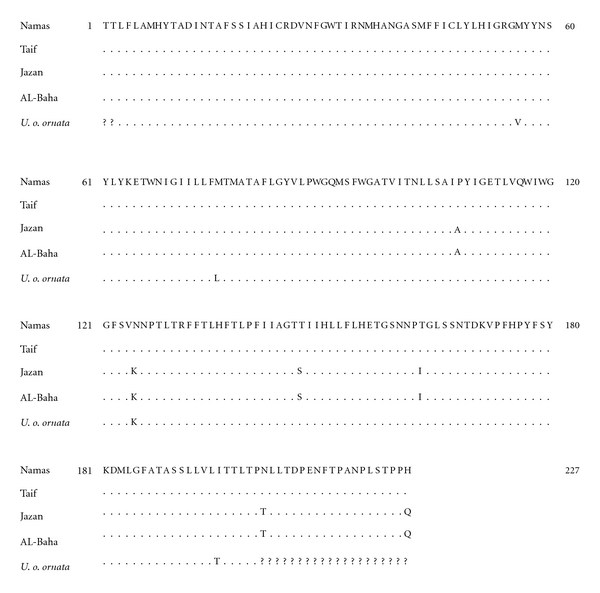
Alignment of partial amino acid sequences of cytb gene for *U. o. philbyi* from different localities. The first sequence is given to that of *U. o. philbyi* from Namas (east Sarawat), and dots for the other sequences denote the identity to this sequence. The sign “?” refers to, nonsequenced portion in *U. o. ornata*.

Including substitutions and insertion-deletion events (indels), the 2.4 kbp region sequenced herein exhibited 84 differences between the individuals on either side of the Sarawat Mountains, of which 22 were characteristic to Jazan samples. The substitutions between the haplogroups of east and west Sarawat were at 25 sites, of which 6 were transversions. Indels are only present in the inserted sequence where only one base deletion was recorded at position 145. Six out of 8 tRNA genes acquired 18 substitutions, of which only 2 transversions were found in T-loop of tRNA^Ile^(C → A) and tRNA^Trp^  (A → C) genes (Table 2). Thirty-four polymorphic sites in the 1035 base pairs representing the complete sequence of the ND2 gene were detected in *U. o. philbyi, *defined the two groups east and west of the Sarawat Mountains. These sites included 29 transitions and 5 transversions. These mutations accounted for 23 synonymous and 11 nonsynonymous substitutions, of which only 1 was characteristic for Jazan (Figure 4). Cytb gene exhibited 6 nonsynonymous substitutions which were characteristic to the west Sarawat haplogroups (Figure 5).

**Figure 6:**
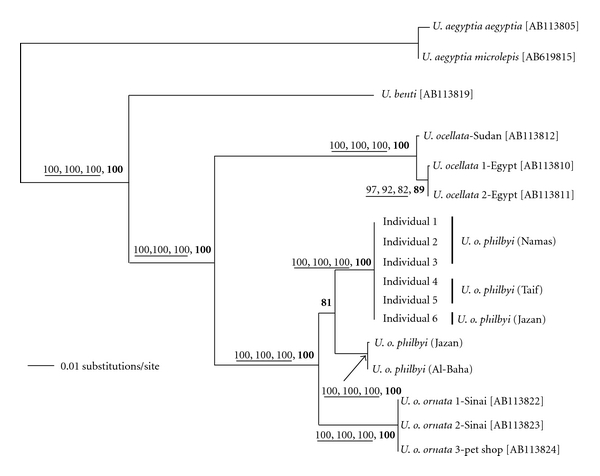
Bayesian tree obtained using 1504 bp of the mtDNA segment of the ND2 gene and seven flanking tRNA genes. Bootstrap values from 1000 replications of heuristic searches by the NJ, MP, and ML methods, respectively, are shown underlined at nodes when they are more than 50%. The bolded values at nodes denote posterior probabilities that were estimated by Bayesian analysis as described in materials and methods. GenBank accession numbers are shown in brackets for the taxa that have not been sequenced in this study.

The overall base compositions of the L-strand of these sequences as accounted by the ML analysis were A: 34.8%; T: 21.4%; C: 31.6%; G: 13.2%.  Figure 6 depicts an ML tree based on the nucleotide sequences of 1504 unambiguous sites aligned in 2 specimens of *U. aegyptia, *3 of* U. ocellata, *3 of* U. o. ornata, *and 8 of* U. o. philbyi*. According to the parsimony criterion, the analysis indicated that 1058 sites are constant, 329 are informative, and 62 are parsimony-uninformative. A single optimal ML tree was found with a negative log likelihood of 4195.16. The same tree topology was obtained by the maximum parsimony and neighbor-joining methods with strong bootstrap probabilities for each nodal relationship as shown on the tree (Figure 6). Bayesian method also supported the tree topology with very strong posterior probabilities at each node. The topological relationship at the basal node of *U. o. philbyi* was only statistically supported by Bayesian analysis (posterior probability = 0.81). The other three analytical methods (MP, NJ, and ML) showed clustering of the west Sarawat group (Jazan and Al-Baha) with the haplogroup of *U. o. ornata *(data not shown); however, this clustering was not trustable because of the weak statistical supports and most probably due to the small sampling size of both subspecies.

As noted above, both subpopulations of *U. ornata philbyi* share the extra 42 bp insertion that *U. ornata ornata* does not have. This qualitative trait provides strong support for the closer relationships of eastern and western *U. ornata philbyi* subpopulations to each other than to *U. ornata ornata*. Moreover, we estimated uncorrected pairwise distances among the different haplotypes (Table 3). Average pairwise distances between eastern and western *U. ornata philbyi* subpopulations were 0.035, which was smaller than that between eastern subpopulations and *U. ornata ornata* (0.038) and that between western subpopulations and *U. ornata ornata* (0.043). These distance data support the conclusion described above.

Individuals of *U. o. philbyi* were shown to be divided into two groups on either side of Sarawat Mountains in spite of the grouping of one Jazan individual with the east Sarawat cluster. A study on the polymorphism of isoenzymes and proteins supported this separation of different haplotypes on either side of the Sarawat Mountains [13]. We believe that collecting more samples and data for *U. o. philbyi* at the subpopulation level could be of great interest to address the status of this subspecies.

Regarding the taxonomic status of the two taxa involved (*ornata* and *philbyi*) we are inclined to interpret the results presented in the present paper as evidence of a differentiation of both taxa on subspecific level. Moreover, the insertion pattern clearly suggests that *U. o. ornata* and *U. o. philbyi* are closely related to each other in relation to *U. ocellata*. There were no obvious differences in the base substitution pattern at the intraspecific and intrasubspecific levels, and the sequence alignment of all studied genes indicated small differences between these two taxa as shown in Figure 5 and Table 3. This is in accordance with the results of Wilms et al. [1], who found the genetic distance between *ornata* and *philbyi* to be 0.7% in the mitochondrial 16S ribosomal RNA gene. This could support the subspecific status of *U. o. philbyi* in relation to *U. o. ornata*.

The mountains from Taif in the middle to Abha in the south parallel to the Red Sea with 1800 km length and over 2 km elevation are known as Sarawat, Aseer, or Hejaz mountains, while the north eastern Jazan area has separate mountains with different names [14–16]. These mountains were found recently to suffer habitat fragmentation and exhibited extinctions for some critically endangered mammals [17]. Such habitat diversity could explain the genetic variation among different haplogroups of *U. o. philbyi* inhabiting these mountains. Further molecular study for more samples from different localities and for samples of *U. o. ornata* from the localities where both subspecies are sympatric could be of great interest in resolving the taxonomic status of both subspecies. We believe that such study is necessary for this endangered and endemic animal in order to be a clue in building a strategy of its conservation.

## Figures and Tables

**Table 1:** Primers used for PCR amplification and/or sequencing. Mixed bases are referred to as R (A, G), Y (C, T), W (A, T), and D (G, A, T).

	Name	Sequence (5′ to 3′)	Source
1:	rND1-3L	CGATTCCGATATGACCAACT	Kumazawa and Endo [4]
2:	rMet-1L	TAAGCTWTYGGGCCCATACC	This study
3:	Uorn-1L	CTGGGCAGGCCTTGAATTA	This study
4:	H6313	CTCTYDTTTGGGGCTTTGAAGGC	Sorenson et al. [5]
5:	rCO1-3H	GTAYAGGGTGCCRATRTCTTT	Kumazawa and Endo [4]
6:	Cytb1-1	TCCAACATCTCAGCATGATGAAA	Kocher et al. [6]
7:	rcytb-1H	GCGTAGGCRAATAGGAAGTATCA	Kumazawa and Endo [4]

**Table 2:** Base substitutions in different tRNA genes sequenced in this study for *U. o. philbyi* on east and west of Sarawat Mountains.

tRNA gene	Base substitution		Location
	East Sarawat	West Sarawat	
Ile (I)	A	G	Anticodon loop
	A	G	Anticodon stem
	C	A	T-loop
	C	T	T-loop

Met (M)	A	G	Acceptor stem
	A	G	Anticodon stem
	T	C	Anticodon stem

Trp (W)	T	C	Acceptor stem
	A	C	T-loop
	G	A	Anticodon stem

Ala (A)	2A	2G	Acceptor stem
	A	G	T -loop

Asn (N)	A	G	D-stem
	T	C	D-loop

Cys (C)	2A	2G	Acceptor stem
	G	A	T -loop

**Table 3:** Average uncorrected pairwise distances between haplotypes from different localities using the ND2 region nucleotide sequences.

	*U. ocellata*	*U. o. ornata*	*U. o. philbyi* (Namas)	*U. o. philbyi* (Taif)	*U. o. philbyi* (Al-Baha)
*U. ocellata*	—				
*U. o. ornata*	0.103	—			
*U. o. philbyi* (Namas)	0.098	0.043	—		
*U. o. philbyi* (Taif)	0.098	0.043	0.000	—	
*U. o. philbyi* (Al-Baha)	0.1001	0.032	0.035	0.035	—
*U. o. philbyi* (Jazan)	0.1001	0.043	0.034	0.034	0.0007
